# Tracking Diurnal Variation in Photosynthetic Down-Regulation Using Low Cost Spectroscopic Instrumentation

**DOI:** 10.3390/s150510616

**Published:** 2015-05-05

**Authors:** Martin van Leeuwen, Robert L. Kremens, Jan van Aardt

**Affiliations:** Rochester Institute of Technology, Chester F. Carlson Center for Imaging Science, 54 Lomb Memorial Drive, Rochester, NY 14623, USA; E-Mails: kremens@cis.rit.edu (R.L.K.); vanaardt@cis.rit.edu (J.A.)

**Keywords:** narrow-waveband, sensor, PRI, photochemical reflectance index, LUE, light-use efficiency, GPP

## Abstract

Photosynthetic light-use efficiency (LUE) has gained wide interest as an input to modeling forest gross primary productivity (GPP). The photochemical reflectance index (PRI) has been identified as a principle means to inform LUE-based models, using airborne and satellite-based observations of canopy reflectance. More recently, low-cost electronics have become available with the potential to provide for dense *in situ* time-series measurements of PRI. A recent design makes use of interference filters to record light transmission within narrow wavebands. Uncertainty remains as to the dynamic range of these sensors and performance under low light conditions, the placement of the reference band, and methodology for reflectance calibration. This paper presents a low-cost sensor design and is tested in a laboratory set-up, as well in the field. The results demonstrate an excellent performance against a calibration standard (*R*^2^ = 0.9999) and at low light conditions. Radiance measurements over vegetation demonstrate a reversible reduction in green reflectance that was, however, seen in both the reference and signal wavebands. Time-series field measurements of PRI in a Douglas-fir canopy showed a weak correlation with eddy-covariance-derived LUE and a significant decline in PRI over the season. Effects of light quality, bidirectional scattering effects, and possible sensor artifacts on PRI are discussed.

## 1. Introduction

Terrestrial ecosystems store approximately 283 to 827 GtC [1,2,3] and play a major role in the exchange of carbon with the atmosphere [4], hence monitoring and understanding forest productivity are of vital importance to understanding global carbon cycles.

A principle variable in the study of land-atmosphere interactions is the gross primary productivity (GPP), which describes the amount of CO_2_ sequestered by vegetation per unit area per unit time, before any losses due to growth and maintenance respiration are accounted for. GPP can be modeled following Monteith [5,6] as the product of incident or absorbed photosynthetically active radiation (PAR) and the light-use efficiency (LUE) which in itself is a function of a wide range of environmental constraints, including temperature, relative humidity, soil moisture, and hydraulic conductivity [7,8]. Over past decennia, a large number of researchers have investigated the dependency of LUE on environmental drivers using both ground-based monitoring networks, as well as remote-sensing techniques, including orbital and airborne imaging.

A significant landmark in the estimation of forest productivity at the stand-level is the eddy-covariance technique that is used to measure fluxes of CO_2_ between ecosystem and atmosphere at approximately 10 ha footprints by measuring high-frequency wind-vector components and CO_2_-mixing ratios using sonic anemometers and gas analyzers [9], from which estimates of LUE can be derived. To date, a network of over 500 permanent eddy-covariance installations covers a wide range of different biomes and is used to create dense time series of productivity estimates across naturally occurring environmental gradients.

Applicable remote sensing techniques have combined the use of spectral and structural information to assess changes in LUE. For example, Gamon *et al.* [10] have demonstrated the use of the Photochemical Reflectance Index (PRI) to explain changes in LUE and attributed these changes to the xanthophyll cycle: a series of carotenoid pigment interconversions that serve to dissipate excess light and protect leaves from photo damage [11]. Most of these measurements were taken at the leaf level and with direct sun illumination. Hilker *et al.* [12] demonstrated the use of shadow fraction to correct PRI observations for bidirectional reflectance effects, enabling stand-level changes in eddy-covariance-derived LUE to be explained with *R*^2^ between 0.6 and 0.8, depending on the range of forest types considered. A recent literature review by Garbulsky *et al.* [13] showed that PRI accounted for 42% of variation in LUE for a wide range of vegetation types considered by including results from over 80 peer-reviewed publications.

Remote sensing studies on interactions between vegetation structure and physiological functioning are, however, limited both in spatial and temporal resolutions and data aggregation over space or time may result in poor understanding of non-linear processes, such as photosynthetic down-regulation; Hence, there is a need to pair airborne and orbital remote sensing observations with dense time-series *in situ* measurements [13].

Recent trends in consumer electronics and engineering design have provided for the ease of building sensor networks that can be deployed to measure the state and dynamics of our environment *in situ*. With a wide range of consumer electronics produced at incrementally lower cost, new research potential is anticipated to investigate non-linear relationships between vegetation functions and structure and to support and extend laboratory-gained knowledge to the field. Garrity *et al.* [14] have introduced and demonstrated a low-cost, narrow-band instrument for measuring PRI at the leaf level and discuss the potential to monitor LUE through deployment of networks of sensors. More recently, Harris *et al.* [15] used data from a narrow-waveband instrument manufactured by Skye Instruments (Llandrindod Wells, UK). This instrument proved capable of observing PRI changes, however, the dynamic range remained limited compared to more expensive spectroradiometers and variability in the position of band center wavelengths was observed across instruments [15]. Moreover, uncertainty remains around the optimal placement of the reference band and methodology used for calibrating reflectance measurements when data are collected within a scattering medium, such as a forest canopy.

In this paper, we present a low-cost, wireless sensor (PRiAnalyze) that was derived from the design provided by Garrity *et al.* [14]. The new instrument has an enhanced dynamic range compared to the previous design, while the wireless communications provide for statically mounted or mobile use, as well as for network deployment and rapid upload of data to a common repository. The proposed design produces digital output and does not require the use of specialized logging equipment. Data for testing are collected within a laboratory set-up and under low-light conditions, as well as during a period of several months in spring and early summer in a coastal Douglas-fir stand. Finally, recommendations for future research are provided.

## 2. Methods

### 2.1. Study Area

Test measurements were made on 3 March 2015, over hydroponic grass (Fresh Patch, Camarillo, CA, USA) that was acclimated to controlled conditions (21 °C) in a laboratory environment at the Chester F. Carlson Center for Imaging Science, Rochester Institute of Technology, Rochester, NY, USA. *In situ* measurements were acquired in a coastal Douglas-fir stand located approximately 50 km south of Campbell River, Vancouver Island, BC, Canada (49°31'11'' N, 124°54'6'' W, 170 m above mean sea level). The site was harvested in 1987 and replanted the following year and currently comprises approximately 75% Douglas-fir, 21% western red cedar and 4% grand fir (*Abies grandis*) [16]. In 2009, the mean tree height, the mean stem diameter at breast height, and leaf area index (leaf area per unit ground area (m^2^·m^−2^)), were 8 m, 8 cm, and 5.3, respectively [16]. The understory is dense and consists of deciduous and evergreen species, the climate is defined as the maritime Coastal Western Hemlock subzone (CWHxm) [17], and the mean annual temperature and rainfall, over the period 1997 to 2012, are 9.6 °C and 1610 mm, respectively [16].

### 2.2. Sensor Design

**Figure 1:**
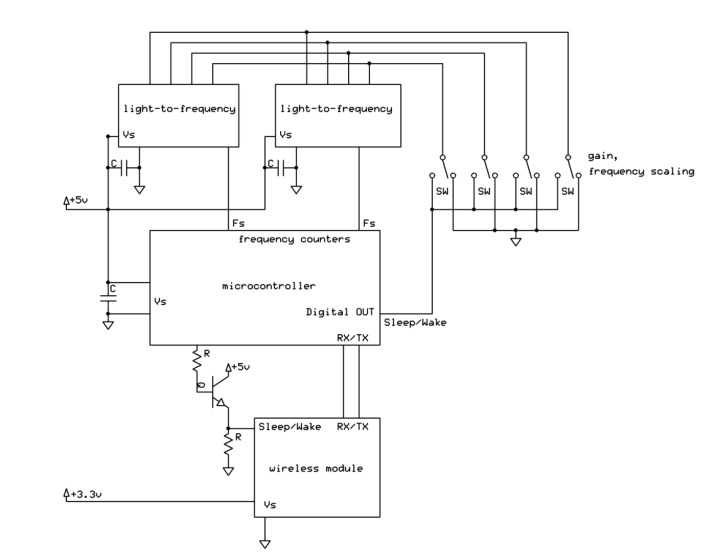
Schematic diagram of the electrical circuit showing the light-sensitive sensors, along with logic circuit and the wireless transmitter.

**Figure 2:**
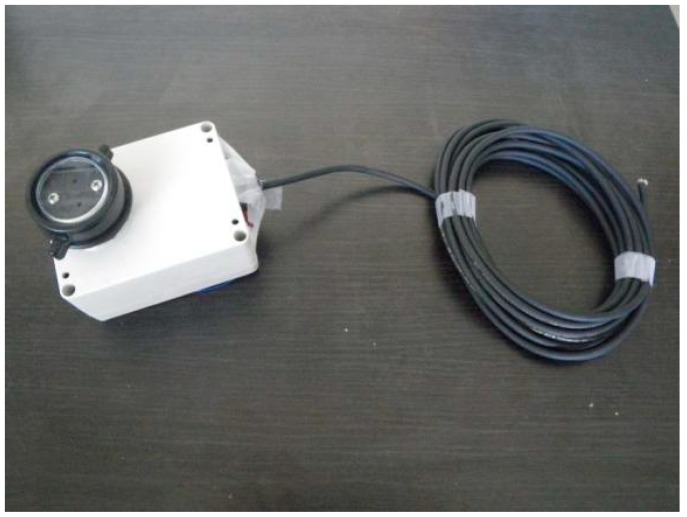
Sensor housing. A cable was used to power the instruments while a wireless link was used for communicating data.

A sensor design that primarily used commercial off-the-shelf (COTS) components was desired. Use of standard plumbing fittings in opto-mechanical assemblies minimized machining costs and the need for specialized machining equipment. The same narrow-waveband interference filters were used as in Garrity *et al.* [14] that feature a 10 nm Full-Width Half Maximum (FWHM). One filter was centered at 532 nm and the other at 568 nm. The electrical circuit of the instrument is based on the TSL-230r (Texas Instruments Advanced Opto-Electronic Solutions Inc., Dallas, TX, USA) programmable light-to-frequency converter that combines a 10 × 10 silicon-photodiode array with a current-to-frequency converter onto a single integrated circuit (IC) [18]. The sensitivity of the IC can be adjusted by setting the photosensitive area to portions of the 10 × 10 array, allowing irradiances in the range 0.01–10,000 μW∙cm^−2^ to be converted to a frequency signal. The TSL-230r was chosen for its low cost, while the frequency output provides ease of read-out by low-cost microcontrollers, and facilitates ease of separation of noise and electromagnetic interference that commonly requires much attention in analog to digital conversion. The IC is packaged in an 8-lead Small Outline IC (SOIC) package for surface mounting. Signals from the TSL-230r were read by an ATMEGA 328p (Atmel Corp., San Jose, CA, USA) 16 MHz microcontroller, that was programmed using the Arduino framework, and was set to integrate frequencies over 500 ms windows. The frequencies were sent wirelessly to a base station using Zigbee modules (Digi International Inc., Minnetonka, MN, USA) [19,20]. The base station was a small Linux-based computer that was connected to the research site’s server, providing Internet access. While the entire system consumes only about 4 W, it was decided to supply the system (including base computer and sensor nodes) with line power, to avoid complexities around battery operation and charging. To reduce energy consumption, the sensors can be put into a cyclic-sleep mode of arbitrary length (e.g., hours, days), where each sleep cycle takes approximately eight seconds. It was anticipated that sunflecks could adversely influence sensor readings and to prevent such adverse effects, a sampling pattern was chosen that was out of phase with reported frequencies of sunflecks that, for temperate forests, has been reported on the order of seconds to minutes [21]. The sensor was programmed to sleep for eight sleep cycles (64 s), followed by a series of three samples, each spaced by one sleep cycle (8 s). Using the average and range of each series of three samples, an indication of representativeness of the sensor reading was obtained and when the difference between any two samples exceeded an order of magnitude or more, the previous series of three samples was deleted. In addition, the sensors can be programmed to remain asleep for prolonged periods of time when irradiance values are below a certain threshold, e.g., at night. This option was not used due to availability of line power at the site and the knowledge gained from receiving data, irrespective of sensor readings, needed to assess the functioning of the wireless data link. Figure 1 and Figure 2 show a schematic of the circuit design, and housing of the sensor, respectively.

The sensor head was created from a 40 mm nominal pipe coupler. A small printed circuit board carrying the photosensitive ICs was mounted onto a 40 mm DELRIN rod of 15 mm in length that had two 12.5 mm holes centered on the photodiode arrays. Following Garrity *et al.* [14] narrow-bandpass interference filters and Teflon^®^ diffusers were installed inside the drilled holes, while stray light arising from the perimeters of the filters was blocked using flat rubber washers. These parts were held into place using thermoplastic glue to avoid the precipitation of solvents onto the optical and photosensitive parts.

### 2.3. Data

The sensor response to varying light intensity was assessed against a calibrated light source (model OL455, Optronics Laboratories, Orlando, FL, USA) that features a tungsten quartz-halogen lamp, a micrometer-controlled aperture that controls the luminance inside an integrating sphere, and a precision silicon detector-filter combination that measures the sphere luminance in foot Lamberts (fL). The calibrated light source provides for a constant color temperature, while the sphere luminance can be adjusted. The raw waveband data from the PRI sensor were recorded simultaneously with luminance readings. From these luminance data, radiance was computed using a conversion table that was provided by the manufacturer, and then only for those tabulated wavelengths that were nearest to the centers of the PRI wavebands (*i.e.*, no interpolation was computed to evaluate the black-body radiation at the waveband centers). To investigate instrument sensitivity within its field of view, the PRiAnalyze was placed under a goniometer that was supplied with a halogen light bulb, enabling incidence angles to be sampled from 0° to 70°.

Laboratory PRI measurements were acquired on 3 March 2015, at the Chester F. Carlson Center for Imaging Science, Rochester Institute of Technology, over (approximately) 60 × 60 cm hydroponic grass turf (Fresh Patch, Camarillo, CA, USA). To accommodate the wide field of view of the PRiAnalyze, mirrors were used to laterally enclose the turf. To ensure that illumination was full spectrum within the narrow wavebands, and that the turf would not be exposed to excessive temperatures, a combination of halogen and LED lighting was used. Luminance was approximately 350 μmol∙m^−2^·s^−1^, measured using an AccuPAR sensor (Decagon Devices, Inc., Pullman, WA, USA). To ensure that the temperature and light spectrum were constant, the light sources were turned on during the entire experiment. To acclimate the turf to darkness, turf was covered with a cardboard lid for 2 h and the lid was then suddenly removed while the PRiAnalyze measured radiance for about 45 min. This sequence of dark-adaptation and illumination was repeated to investigate if any potential reflectance changes were reversible.

Field data were acquired between 6 March 2013 and 14 June 2013 from a scaffolding tower that provided access to a Douglas-fir canopy. The sensor was fixed using a wooden boom that was attached to the top of the scaffolding tower at around the 7-m height, and the sensor head was placed about 60 cm above a conifer whorl using a nadir field of view. This location was chosen to minimize the occurrence of sunflecks in the field of view, while the sensor was still easily accessible from the tower. Moreover, sensors at this height can more easily be mounted to stems, with potential to reduce installation costs. Data were periodically downloaded to the University of British Columbia and examined for irregularities.

Eddy-covariance data, along with meteorological records of total photosynthetically active radiation (PAR), temperature, and relative humidity, were obtained from the Land and Food Systems Department (The University of British Columbia, Vancouver, BC, Canada) for the study site and period. EC-derived estimates of stand-level gross primary productivity, GPP, were calculated as daytime ecosystem respiration, $R_{e}$, minus daytime Net Ecosystem Exchange, NEE. The daytime $R_{e}$ was estimated using the exponential relationship between measured nighttime NEE and soil temperature at 2 cm depth [22] and NEE was calculated as the sum of above-canopy EC-measured CO_2_ flux. See Morgenstern *et al.* [23] and Barr *et al.* [24] for further details on methodology used in processing the eddy-covariance data.

## 3. Processing

The PRI sensor data were quality checked and instrument-caused data outliers were removed. These data outliers were one or several orders of magnitude larger than the observed trends in the signal and beyond the instrument calibrated range, hence a low-pass filter, with a cut-off of values above 1.2 × 10^−5^ W·cm^−2^·sr^−1^, sufficed to remove all outliers and resulting data gaps were then filled using linear interpolation. Moreover, data outages caused some missing data and a decision was made to include days with full coverage only (including, March: 6; April: 18–21, 25–27; May: 2–31; June: 1–30; July: 1–13), thus ensuring that only full diurnal data sets were used in the analysis. Using the filtered data set, PRI time series were computed using calibrated radiance data as:

$$\text{PRI}=\frac{I_{532}-I_{568}}{I_{532}+I_{568}}$$

The filtered, gap-filled PRI data were smoothed using a window size of 90 data samples. All data were stored in a Postgres database and daily and half-hourly averages were queried and temporally matched to records of eddy-covariance GPP and PAR. Light use efficiency (LUE, units: μmol·C·μmol^−1^ photons) can be expressed as the ratio of GPP to either absorbed [25] or incident PAR [26]. The latter form requires no knowledge of canopy architecture and was adopted in this study for the sole purpose of demonstration, acknowledging the need for a common basis [27].

## 4. Results and Discussion

### 4.1. Calibration

**Figure 3:**
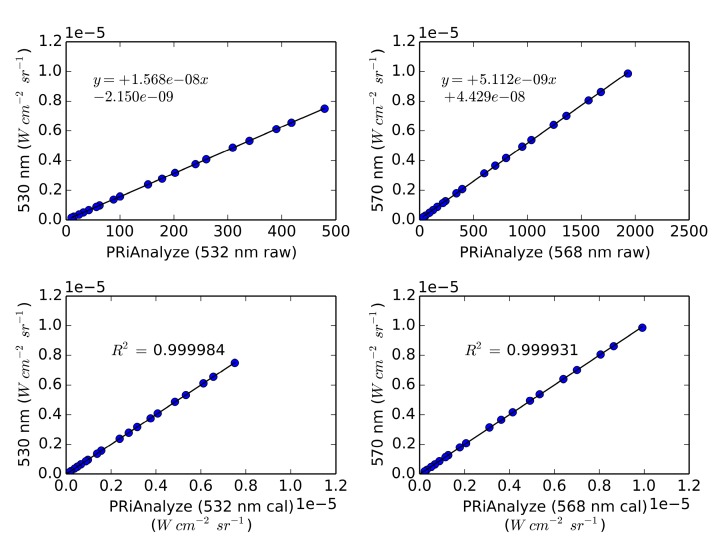
Correlations between the PRiAnalyze waveband data (*x*-axis) and the calibrated light source (*y*-axis).

Figure 3 shows the correlation between the calibrated light source and the PRI sensor. The plots demonstrate the linearity of the sensor response to incident radiance across a wide dynamic range and show correspondence close to the 1:1 line and a high coefficient of determination.

**Figure 4:**
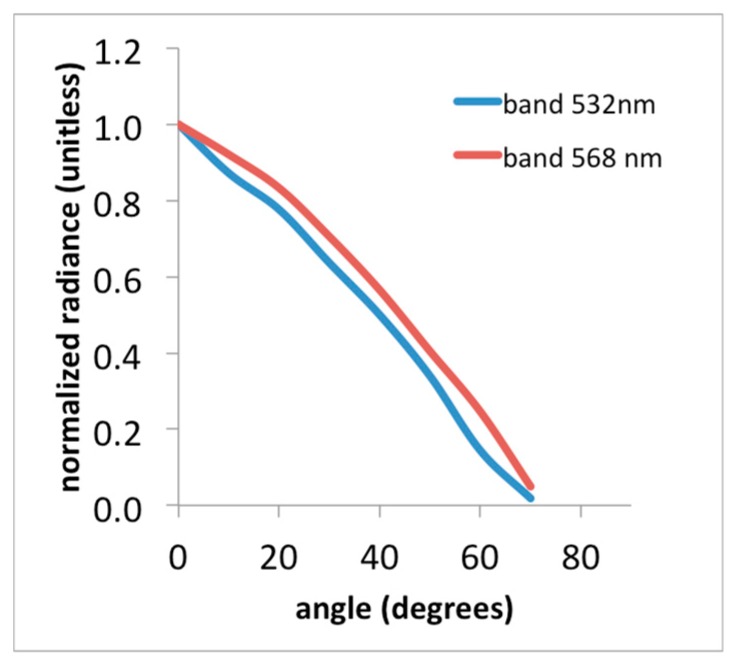
Results from goniometer measurements show waveband response across a range of incidence angles. Note the sensor field of view is approximately 140°.

The angular response of the PRiAnalyze was assessed using the goniometer measurements and radiance values normalized by the radiance at the normal-incidence angle exhibited a shape between linear and cosine (Figure 4). At an incidence angle of 70° from the normal, the 532 nm waveband reached zero, while readings from the 568 nm waveband reached a minimum. No larger range of angles was investigated, as the goniometer’s range was limited to 70°. Deviations from a cosine shape can be explained by shadowing from the rim of the sensor head and the rubber washers enclosing the Teflon^®^ diffusers that restrict the field of view to a range smaller than 2π·sr. As a result, the sensor field of view (FOV) was approximately 140°. Effects of angular incident radiation on bandwidth and center wavelength of the interference filters were not investigated in this study, and potentially can explain additional discrepancies between the angular responses of the two channels.

### 4.2. Laboratory Data

**Figure 5:**
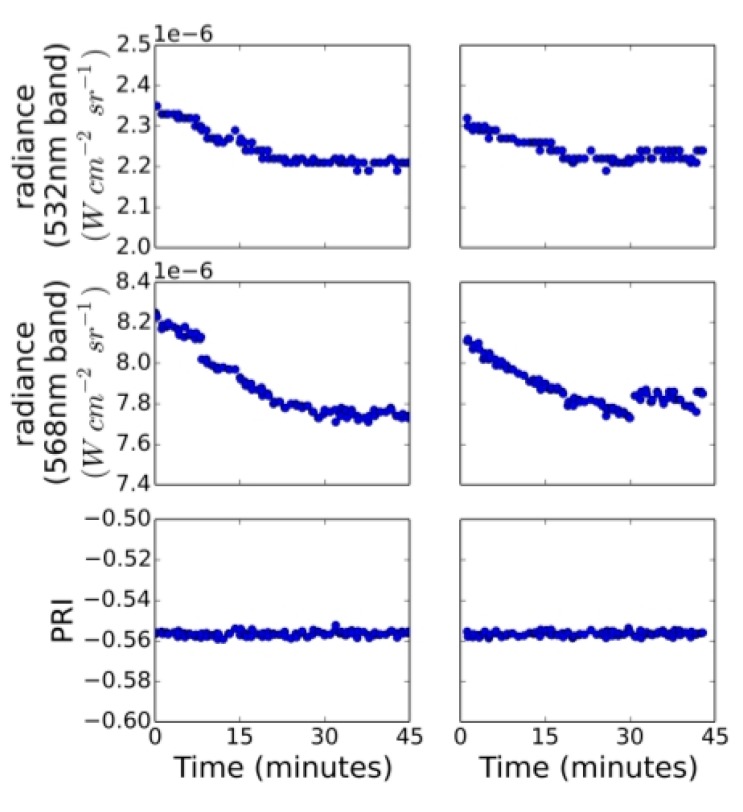
Change in reflected radiance measured in the two PRI wavebands over time as turf was exposed to approximately 350 μmol·m^−2^·s^−1^. First series of measurements are shown in the first column. The second series of measurements were acquired after acclimating the turf to darkness for two hours and are shown in the second column.

Waveband measurements of hydroponic grass showed a marked decrease in radiance values as the illumination changed from darkness to lit (Figure 5). These reflectance changes were observed over a period of about 30 min; however, in contrast to earlier findings [10] these changes were observed in both the reference and signal bands.

### 4.3. Field Data

**Figure 6:**
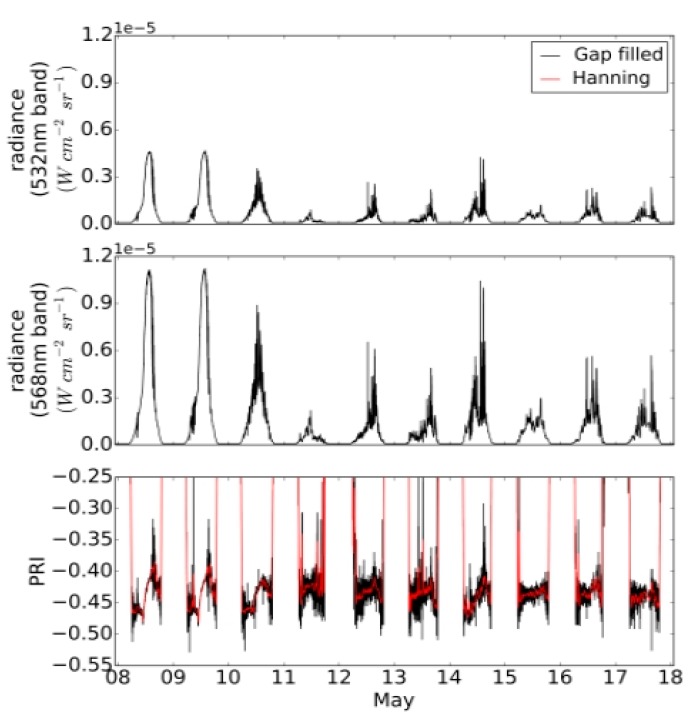
Graphs showing the calibrated radiance data in the 532 nm and 568 nm wavebands and the photochemical reflectance index (PRI). Dates shown are in the Pacific Standard Time zone (*i.e.*, UTC-08:00).

Figure 6 shows a sample of data from the PRI sensor for days in May. Top and middle panels show the signal and reference band, respectively, while the bottom panel shows the filtered, gap-filled and smoothed PRI signals, *i.e.*, using the 90-samples window. The diurnal PRI signals show a marked recurrent pattern for two sunny days of 8–9 May, and a much smaller variation for measurements obtained from cloudy or partially cloudy days of 11–17 May. The measurements show, moreover, a strong performance under low light conditions as indicated by the steep rise and fall of the PRI signal at dusk and dawn, respectively, and constancy of the PRI signal under cloudy conditions observed for, e.g., 15 May.

**Figure 7:**
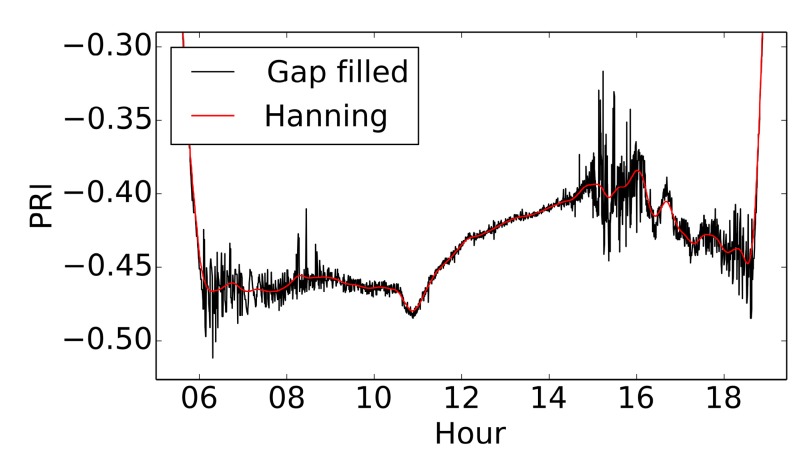
PRI signal shown for 8 May 2013. Times shown on the *x*-axis are in the Pacific Standard time zone.

Figure 7 shows a detailed view of the PRI signal for 8 May 2013. In examination of the diurnal PRI signals for sunny days, we observed a behavior that was inverted from results reported in literature. While we expected PRI to decrease with increasing irradiation, we observed a small decrease around 11 a.m. followed by a large increase, and then a decrease again towards the end of day. It is likely that this opposing result is caused by factors other than xanthophyll-induced down-regulation. For example, off-axis behavior of the interference filters, which can influence both bandwidth and position of the center wavelength, can lead to a reduction in the ability to discriminate radiance differences between the signal and reference wavebands. This is an important effect that warrants further study, even though similar previous studies have not explicitly mentioned or investigated this artifact. Other factors impacting the observed signal include changes in the spectral quality of incident radiation, and bidirectional reflectance effects within the sensor FOV as the irradiation conditions changed from diffuse to direct sun and the sun angle changed [25].

### 4.4. Daily EC-Derived LUE vs. Narrow-Waveband-Derived PRI

**Figure 8:**
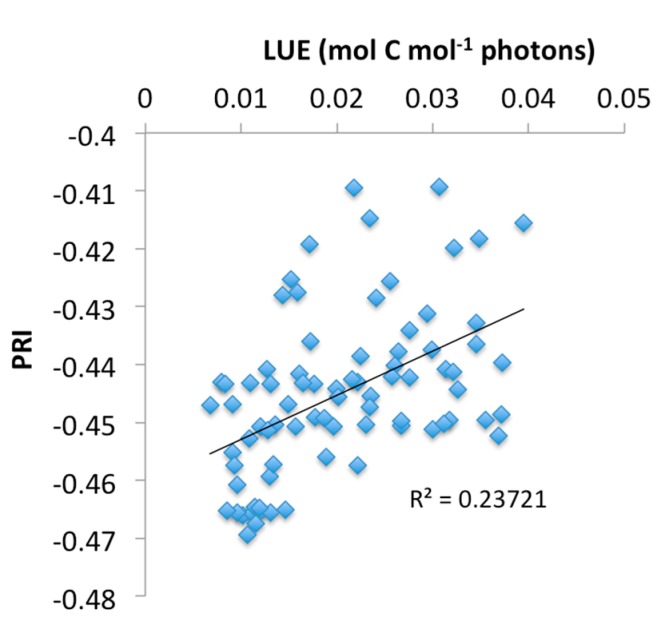
Scatter plot showing the relationship between the daily averaged photochemical reflectance index (PRI) and daily-averaged light use efficiency (LUE) that was derived with the eddy-covariance technique, and selected for times between 8 a.m. and 4 p.m. Pacific standard time.

**Figure 9:**
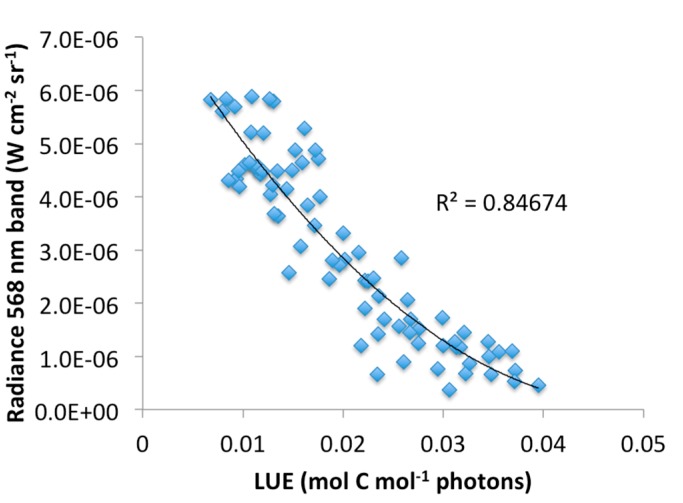
Relationship between upwelling radiance, as measured by the 568 nm band, and light-use efficiency (LUE), as derived using the eddy-covariance technique.

**Figure 10:**
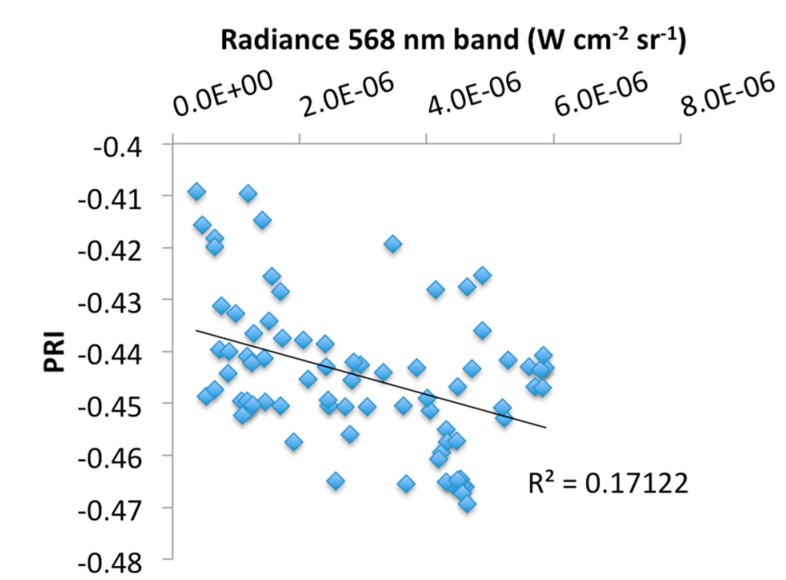
Relationship between the photochemical reflectance index (PRI) and upwelling radiance measured in the reference band (568 nm).

Figure 8 and Figure 9 show the relationships between eddy-covariance estimates of stand-level LUE against PRI observations (Figure 8), and calibrated radiance data from the 568 nm band (Figure 9). Data points represent daily averages for the period March to July for selected times between 8 a.m. and 4 p.m. PST. In general, high PRI and LUE values were found for cloudy days with low irradiance, while sunny days related to low PRI values and low LUE, in agreement with earlier findings; however, a much smaller correlation was found than previously has been reported for a similar forest environment [12]. The moderate correlation can partly be explained from marked differences in scale between the EC-derived LUE and the PRI measurements and restricted availability of data covering the growing season (*i.e.*, a larger down-regulation of photosynthesis is expected to occur in the dryer and hotter months of July and August). Changes in radiance data from the 568 nm band correlated closely with LUE, as expected from the definition of LUE being the ratio of GPP and incident PAR, and a second-order polynomial fit resulted in an explained variation of *R*^2^ = 0.85. Weak correlations were found between PRI and EC-derived LUE estimates (Figure 8) and between PRI and upwelling radiance in the 568 nm band (Figure 10); however, the latter correlation was the weakest. Further research is needed to validate these results against fluorescence measurements and gas-exchange measurements performed at scales that are consistent with the PRI observations.

**Figure 11:**
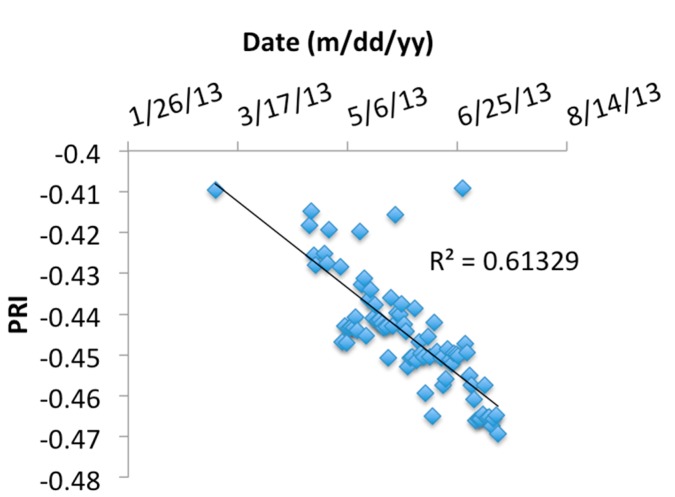
Change of the photochemical reflectance index (PRI) over time. Two largest discrepancies between observations and model fit represent days with lengthy periods of low-light conditions in mornings that caused visible noise in PRI data. *R*^2^ = 0.8 would have been achieved by selecting a shorter time span, e.g., 10 a.m. to 2 p.m.

In addition to correlations between LUE and PRI, trends in PRI over time were examined and exhibited a strong linear decrease in PRI over the period March to July (*R*^2^ = 0.6), shown in Figure 11. The two largest errors in the model fit were caused by days with a prolonged period of low-light conditions and for these days some visible noise was observed in the PRI data. Restricting the time-of-day selection between 10 a.m. and 2 p.m. improved the model fit to *R*^2^ = 0.8 (data not shown). The decrease in PRI is likely linked to phenological changes, including chlorophyll:carotenoid ratio changes [13] and growth, as well as changes in irradiation quality and sun angle effects that could not be further examined using the collected data sets.

## 5. Conclusions

A design was presented in this paper for a low-cost narrow-waveband sensor for the collection of PRI data. The design and low cost of the sensor presented in this paper facilitate the unattended deployment into larger networks to cover broad scales in a manner that fits contemporary data needs and budget constraints. Such networks hold potential value for the calibration and validation of remote sensing data. For example, fine-spatial, *in situ* measurements of PRI may be an important source of information for the assessment of algorithms used to subdue atmospheric and surface BRDF effects present in airborne and tower-based data or to gain a better understanding of fine structure-function relationships.

Calibration of the instrument using a calibrated light source resulted in a high correlation coefficient and the instrument response was very linear. Under moderately low-light conditions, the instrument performed well and showed reductions in turf reflectance in both wavebands, when the turf was illuminated with a combined LED/Halogen light of approximately 350 μmol photons m^−2^·s^−1^, representing conditions such as may be encountered outdoors on a cloudy day; However, no significant change in PRI was noticed. Using PRI observations of the upper strata of a Douglas-fir canopy for times-of-day between 8 a.m. and 4 p.m., about 23% of variation in EC-derived daily stand-level LUE estimates were explained. The finding is significant given the difference in scale between the EC and PRI instruments and limited temporal coverage over the season. However, using the data available, diurnal trends in the PRI signal could not be well explained, and some are likely due to factors not related to xanthophyll-induced down-regulation of photosynthesis, including BRDF effects and changes in incident light quality. Further research is needed to investigate the sensitivity of the bandwidth and center-wavelength of the interference filters to varying incidence angle. The latter may require use of low-cost optics to improve the sensor cosine response and collimate incoming radiation before passing through the interference filters. Variations in bandpass center wavelength across sensors of the same make and model have also been noted by Harris *et al.* [15]; however, investigations as to how this issue may be resolved for low-cost acquisition of PRI data have not been found so far.

A word of caution is appropriate for interpretation of the PRI index. Unlike other normalized difference vegetation indices, such as the Normalized Difference Vegetation Index (NDVI) that is used to assess vegetation structure or vigor from canopy reflectance data, both channels of the PRI index are sensitive to changes in photosynthetic down-regulation. In fact, since xanthophyll-induced down-regulation of photosynthesis relates to changes in PAR, observations in a single waveband can also explain significant portions of the total amount of variation in photosynthetic down-regulation. Moreover, an incorrect calibration can correlate with EC-derived LUE, for reasons other than actual reflectance changes caused by (de-)epoxidation of xanthophyll and associated conformational changes. Ideally, calibrations should be conducted frequently using calibration standards and results should be quality checked to ensure PRI remains constant with changing illumination intensity at a constant color temperature. Other concerns on the use of PRI are the potential influence of phenology [13,28] and physiological mechanisms, besides the xanthophyll cycle, that result in a photosynthetic down-regulation, such as chloroplast avoidance movement [29] and chlorophyll content [30].
